# Language Experience Changes Audiovisual Perception

**DOI:** 10.3390/brainsci8050085

**Published:** 2018-05-11

**Authors:** Viorica Marian, Sayuri Hayakawa, Tuan Q. Lam, Scott R. Schroeder

**Affiliations:** 1 Department of Communication Sciences and Disorders, Northwestern University, Evanston, IL 60201, USA; sayuri.hayakawa@northwestern.edu; 2 Department of Psychological Sciences, Loyola University, New Orleans, LA 70118, USA; tlam@loyno.edu; 3 Department of Speech-Language-Hearing Sciences, Hofstra University, Hempstead, NY 11549, USA; Scott.R.Schroeder@hofstra.edu

**Keywords:** speech perception, bilingualism, multisensory integration, McGurk effect, language

## Abstract

Can experience change perception? Here, we examine whether language experience shapes the way individuals process auditory and visual information. We used the McGurk effect—the discovery that when people hear a speech sound (e.g., “ba”) and see a conflicting lip movement (e.g., “ga”), they recognize it as a completely new sound (e.g., “da”). This finding that the brain fuses input across auditory and visual modalities demonstrates that what we hear is profoundly influenced by what we see. We find that cross-modal integration is affected by language background, with bilinguals experiencing the McGurk effect more than monolinguals. This increased reliance on the visual channel is not due to decreased language proficiency, as the effect was observed even among highly proficient bilinguals. Instead, we propose that the challenges of learning and monitoring multiple languages have lasting consequences for how individuals process auditory and visual information.

## 1. Introduction

As we go about our day, our minds are constantly integrating incoming sensory inputs. In its extreme form, sensory integration can result in rare but fascinating synesthetic experiences such as seeing yellow when hearing “o” or tasting the month of June [1,2]. While most of us do not experience radical cross-modal perceptions, some form of multisensory integration is found even in infants [3] and non-human primates [4], speaking to how naturally and seemingly effortlessly we bind different sensory inputs. It might be counterintuitive then, to think that such a fundamental perceptual process could be influenced by our experiences. We propose that it can. To explore this idea, we turn to language as a source of experience, and ask whether the use of two languages (i.e., bilingualism) modulates audio-visual integration. In other words, can being bilingual change how and what we hear?

It has been well documented that what we smell affects our perception of flavor [5]. But other, more unexpected interactions have been observed as well. Listening to smooth music can affect the perceived creaminess of chocolate [6], and what we see with our eyes can alter our perception of the shape, texture, and even temperature of what we feel with our hands [7]. What we hear is often profoundly affected by what we see [8,9]. One of the most dramatic demonstrations of how different modalities influence each other is the well-known McGurk effect [10]. The McGurk effect refers to a curious phenomenon whereby what we see changes what we hear. Participants are presented with auditory stimuli such as /ba/, but see the speaker saying an incongruent sound such as /ga/. Rather than hearing the actual auditory stimulus (/ba/), which is articulated at the front of the mouth, or the actual visual stimulus (/ga/), articulated at the back of the mouth, individuals will often perceive a fused sound such as “da”, which is articulated somewhere in between. This highly robust effect demonstrates that what we hear is not independent of what we see, and that the whole percept can be different than the sum of its parts.

While this type of audiovisual integration occurs naturally and frequently, the extent to which we rely on visual information to guide auditory input can depend on contextual and individual factors. Unsurprisingly, we rely on visual input more when the auditory input is unclear, such as when the audio is degraded or under noisy conditions. For instance, visual lip-reading can help clarify degraded speech sounds [11]. As a result, individuals are more likely to experience McGurk effects when auditory input is less intelligible [12,13,14]. Bilinguals may thus experience greater audiovisual integration when utilizing their less dominant tongue, as less familiarity with a language induces greater reliance on the visual channel in order to make sense of auditory input [15,16,17,18]. However, we propose a more radical notion regarding the effect of bilingualism on audiovisual integration. We propose that even after language competence has been reached, the early challenges of learning and managing multiple languages may alter the way that individuals perceive and integrate audiovisual stimuli. If so, it would suggest that the way we process sensory information can be influenced not only by our immediate environment, but also by our past experiences.

Bilingualism has been shown to have long-term consequences for a range of cognitive processes. For instance, research shows that bilingual experience can increase cognitive control [19,20], enhance meta-linguistic awareness [21,22], delay the effects of Alzheimer’s disease in older adults [23,24], and improve perspective-taking ability in children [25,26]. While such examples illustrate the various benefits of bilingualism, there are some costs as well. Many previous studies have reported that bilinguals using their second language often have more difficulties than monolinguals in comprehending speech in noisy environments [27,28]. One potential reason for this speech-in-noise deficit is less experience with words in each language. Because many bilinguals split their time between two languages, the frequency of any given word is reduced for bilinguals in comparison to monolinguals (consistent with the frequency-lag or weaker-links hypothesis; [29,30]). A second potential reason is interference from words in a bilingual’s other language. Because bilinguals access words from both languages even when using only one language, competition from words in the non-target language may impede access to the target word (a cross-linguistic interference hypothesis [31,32]). Similar interference has been observed at the phonemic level: when presented with nonnative speech-sounds, listeners are biased by the perceptual boundaries of their native tongue [33,34]. As a result, bilinguals can suffer a deficit when interpreting stimuli not only at the word level, but at the phonemic level as well [35]. Lastly, being raised in a bilingual household can uniquely tune infants towards processing the speech of bilingual adults [36]. Bilingual infants may therefore rely on cues other than auditory input to help process less familiar monolingual speech. 

The complex task of learning multiple languages could thus shape bilinguals’ strategies for speech comprehension. Navarra and Soto-Faraco [37] find that bilinguals’ comprehension of speech is greatly enhanced by attending to corresponding visual stimuli. This could lead to a general tendency to rely more on the visual channel. In line with this hypothesis, it has been found that even prelinguistic infants gaze more at the mouth area of speakers if raised in a bilingual environment [38]. It may therefore be the case that bilinguals attend more to visual information, either due to on-going deficits in comprehending speech, or to early challenges that result in habits of perceptual processing that persist even after full proficiency has been attained. 

In the current study, we present participants with auditory and visual stimuli that are either congruent or incongruent with each other. Participants were tasked with identifying the auditory speech-sound with and without accompanying visual input, as well as with or without noise. Speech-sounds were produced by a native English speaker. We assessed the degree of audiovisual integration by examining the frequency of McGurk-like percepts whereby incongruent audiovisual stimuli (e.g., hearing /ba/ and seeing /ga/) are heard as a fused sound (e.g., /da/). We also assessed basic proficiency at identifying speech-sounds by looking at the proportion of correct identifications for auditory input when it is presented alone as well as when it is paired with congruent visual stimuli (e.g., hearing and seeing /ba/). Critically, we compared three groups of individuals: monolingual native English speakers (*N* = 17), early Korean-English bilinguals (*N* = 18) with self-reported English proficiency that is equal to the monolinguals, and late Korean-English bilinguals (*N* = 16) with English proficiency that is lower than both monolinguals and early bilinguals. 

Due to the relatively complex task of acquiring multiple languages, bilinguals may rely on visual information to a greater degree than monolinguals, resulting in increased audiovisual integration. Greater reliance on the visual channel during bilingual speech comprehension and a bilingual increase in audiovisual integration due to greater difficulty comprehending speech would be analogous to previous findings that noisy listening conditions increase the likelihood of experiencing McGurk effects [12,13,14]. This enhancement may result from either on-going difficulty with perceiving foreign language speech sounds, or from habits of processing that persist even after full proficiency is attained. In the case of the former, we would expect to see a larger McGurk effect for bilinguals who acquired English later in life, but not necessarily for those who acquired it early. Additionally, we should observe that greater susceptibility to the McGurk effect is accompanied by increased difficulty in perceiving speech-sounds in the audio-only and congruent audiovisual conditions. On the other hand, if bilingual experience has lasting effects on perceptual processing that persist beyond difficulty comprehending speech-sounds, we should observe a greater McGurk effect for both early and late bilinguals. Importantly, this would not necessarily be associated with greater difficulty in comprehending speech-sounds during audio-only and congruent audiovisual trials, particularly for early learners. 

## 2. Materials and Methods

### 2.1. Participants

Fifty-one young adults with normal hearing participated in this study (see Table 1 for participant demographics). Participants belonged to one of three groups: English-speaking monolinguals, early Korean-English or English-Korean bilinguals, and late Korean-English bilinguals. Early bilinguals varied in whether English or Korean was acquired first, but both English and Korean were learned before age 7. On average, early bilingual participants were comparably proficient in their two languages, as measured with the Language Experience and Proficiency Questionnaire [39]. Late bilinguals all spoke Korean as a first language and learned English as a second language after the age of 7. Late bilingual participants were slightly, but not significantly, more proficient in Korean than in English. Monolingual participants were native speakers of English who listed either limited or no proficiency in languages other than English. Between-group comparisons of proficiency are presented in Table 1.

### 2.2. Design

Stimuli consisted of both audiovisual and auditory-only speech syllables (e.g., /ba/). The audiovisual stimuli were presented within-subject in (1) quiet and noisy auditory conditions; (2) congruent and incongruent audiovisual conditions. In the quiet condition, stimuli were presented without background noise, while in the noise condition, stimuli were presented amid unintelligible speech from six different speakers (i.e., six-talker babble). In the congruent condition, the auditory and visual input matched, while in the incongruent condition, the auditory input was a sound produced with the lips (e.g., /ba/ or /pa/) and the video input was of a sound produced at the velar position (e.g., /ga/ or /ka/). The audiovisual (AV) stimuli were used to assess the extent to which individuals attended to the visual information to perceive auditory inputs. We also included auditory-only stimuli which were accompanied by a static photo of the speaker, also in quiet versus noise conditions. As there is no visual speech presented in the auditory-only condition, congruent versus incongruent could not be manipulated for the auditory-only stimuli. The auditory-only conditions allowed us to test for a standard speech-in-noise deficit and served as a baseline for the AV congruent and incongruent conditions.

### 2.3. Materials

Syllables were produced by a female native speaker of English. There were six syllables presented: /ba/, /da/, /ga/, /pa/, /ta/, and /ka/. Auditory intensity of each sound and video file was normalized at 60 dB peak to peak. The babble noise was presented at 70 dB sound pressure level (SPL; +10 dB compared to the stimuli). Speech sounds and noise were delivered through disposable earbuds. The use of earbuds ensured a consistent proximity between the subject and the source of the auditory stimuli, and limited exposure to unintended ambient sounds. Visual stimuli were displayed using a 27-inch iMac computer using MATLAB. The screen resolution on the computer was set at 2560 × 1440 pixels. The visual stimuli were rendered at 640 × 480 pixels with a digitization rate of 29.97 frames per second (33.33 ms/frame), and the stereo stimuli were digitized at 44.1 kHz with 32-bit resolution. Incongruent videos were created by aligning the consonantal burst of the audio file (e.g., ‘ba’) with the consonantal burst of the underlying audio portion of the video file (e.g., ‘ga’) within ±5 ms, as described by van Wassenhove, Grant, and Poeppel [40,41]. Auditory-only stimuli were obtained by extracting the audio from the videos. Instead of a video, participants saw a static image of the speaker with her mouth closed while only the auditory stimulus was played. All stimuli were generously provided by Dr. Ken Grant from the Walter Reed National Military Medical Center.

The /ba/ and /pa/ auditory trials served as critical trials. When paired with visual stimuli /ga/ or /ka/, participants may perceive the actual auditory input (/ba/ or /pa/), the actual visual input (/ga/ or /ka/), or a fused percept of the two (/da/ or /ta/). To ensure that auditory stimuli other than /ba/ or /pa/ were potential candidates for selection, trials with a true /da/, /ta/, /ga/, and /ka/ auditory stimulus were added as filler trials. For audio-only trials, each syllable was presented ten times in each of the two conditions (quiet and noise), leading to 40 critical trials and 80 filler trials. As with audio-only trials, audiovisual critical syllables were presented ten times in each of the four conditions (including congruent and incongruent trials) for a total of 80 audiovisual critical trials. For audiovisual filler trials, because incongruent trials with true auditory stimuli of /ba/ or /pa/ may lead speakers to respond with the video-consistent syllable (/ga/ and /ka/) or the fused syllable (/da/ and /ta/), there would be fewer perceived instances of /ba/ or /pa/. To compensate for this difference and create the sense of a more equal distribution across the six possible syllables, there was a 1:1 ratio of critical trials to filler trials in the audiovisual condition as opposed to the 1:2 ratio present in the audio-only condition. This led to a total of 80 filler trials in the audiovisual condition, resulting in a total of 280 trials overall.

### 2.4. Procedure

**Figure 1:**
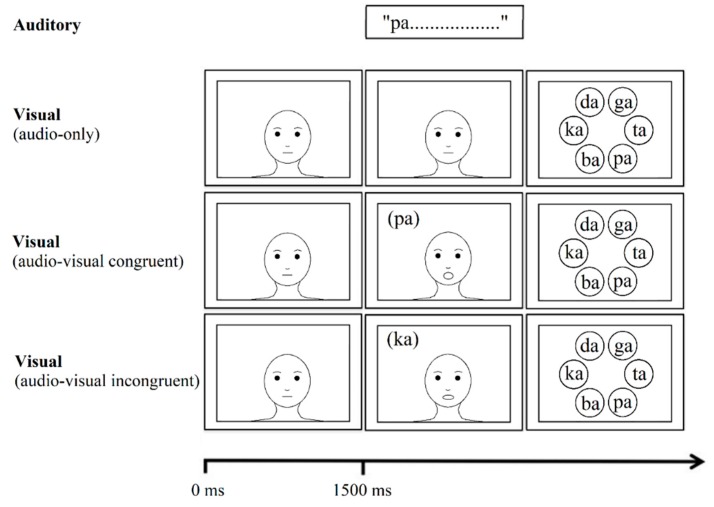
A depiction of an example trial. In the auditory modality, the target speech sound was played 1500 ms after trial onset. In trials with noise, the babble began at trial onset and continued until the end of the speech sound. In the visual modality, at trial onset, a motionless face was shown for 1500 ms. Then, participants either continued to see a motionless face (auditory-only trials), saw a face producing a matching sound (audio-visual congruent trials), or saw a face producing a mismatching sound (audio-visual incongruent trials). After the speech sound was finished playing, participants saw a six-item forced choice display and had to click on the speech sound that they heard.

After completing the Language Experience and Proficiency Questionnaire (LEAP-Q [39]), participants began the audiovisual experiment. At the start of a trial, participants first saw a motionless face. In audiovisual quiet trials, the motionless face was present for 1500 ms before beginning to produce the target speech syllable which lasted approximately 500 ms, followed by 500 ms of silence. The visual stimulus thus remained on screen for 2500 ms. On noise trials, babble noise began playing at trial onset beginning 1500 ms before the beginning of the target speech syllable and lasting for the duration of the stimulus presentation. In audio-only trials, the video was replaced by a still image. Then, participants were presented with a six-item forced choice display and had to indicate the sound they heard. After indicating their response, the next trial began. See Figure 1 for a visual display of a trial.

The 280 trials were split into ten blocks. After every block, participants were given a break of approximately two minutes before continuing with the experiment. At the halfway point of the experiment, participants were given a longer break (5–15 min). 

### 2.5. Data Analysis

To examine the effects of language group and English proficiency on audiovisual integration, we began by running an ANOVA under noisy and quiet conditions with each participant’s arc-sine transformed proportion of McGurk responses as the dependent variable. Language Group (monolingual, early bilingual, late bilingual) and self-reported English Proficiency were entered as between-subject predictors. As a robustness check of the Language Group effect, we followed up with a generalized linear mixed effects analysis (lme4 in the R environment) that accounts for trial-level variance. Each trial response was coded as either McGurk or not-McGurk and entered as a binary outcome variable, with a fixed effect of Language Group and random intercepts for subject and item. Language Group was contrast coded to first compare bilinguals (combined late and early) against monolinguals, followed by early versus late bilinguals. 

Next, we examined the effects of language group and proficiency on speech perception ability using a repeated-measure ANOVA with each participant’s accuracy score as the dependent variable. Language Group and self-reported English Proficiency were entered as between-subject predictors, while AV status (i.e., audiovisual congruent versus audio-only) was entered as a within-subject predictor. As with the McGurk analysis, this was followed by a generalized linear mixed effects regression as a robustness check. In this case, accuracy on each trial was entered as the dependent variable, with Language Group (contrast coded to compare bilinguals to monolinguals and then early to late bilinguals) and AV status (sum coded) as fixed effects, and random intercepts for subject and item. 

### 2.6. Data Availability

The datasets generated and/or analyzed during the current study are available from the corresponding author on request.

### 2.7. Use of Human Participants

The experiment was carried out with the approval of Northwestern University’s Institutional Review Board following approved guidelines and regulations. Informed consent was obtained from all participants prior to participation. 

## 3. Results

### 3.1. The McGurk Effect

#### 3.1.1. Quiet Condition

**Figure 2:**
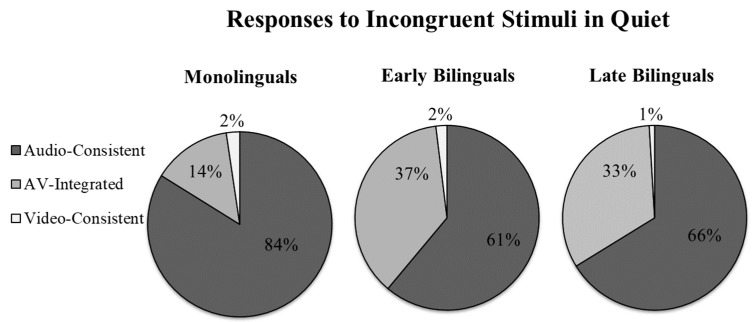
Percentage of auditory-consistent responses, audiovisual (AV)-integrated responses, and video-consistent responses to incongruent stimuli in the Quiet condition.

**Figure 3:**
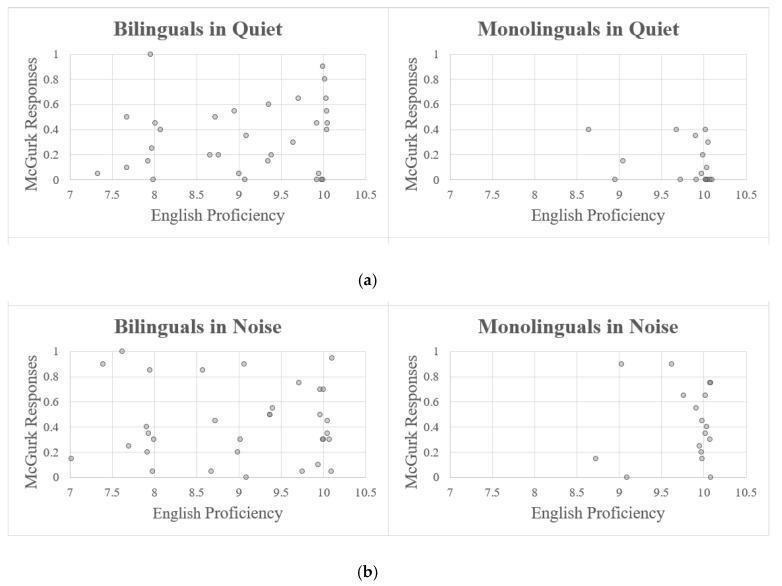
Relationship between English proficiency (jittered by a random value between ±0.1) and proportion of McGurk responses under quiet (**a**) and noisy conditions (**b**).

The ANOVA revealed a significant main effect of Language Group, with bilinguals experiencing more McGurk effects (i.e., AV-integration) than monolinguals (F(2,44) = 4.08, *p* = 0.024; Figure 2). There was no main effect of English proficiency (F(1,44) = 0.54, *p* = 0.465; Figure 3), and no interaction with language group (F(2,44) = 0.355, *p* = 0.703). We followed up by conducting pairwise comparisons of each of the three language groups with Bonferroni corrections for multiple comparisons (*p* < 0.016 was considered significant). As expected, late bilinguals experienced significantly more AV-integration than monolinguals (F(1,28) = 6.97, *p* = 0.013), with no effects or interactions with English proficiency (both *p* > 0.4). Critically, the same pattern was observed with early bilinguals (F(1,31) = 6.60, *p* = 0.015), with no effects of English Proficiency or interactions (both *p* > 0.2). Lastly, early and late bilinguals did not differ from each other (F(1,29) = 0.429, *p* = 0.881), nor was there a main effect or interaction with English Proficiency (both *p* > 0.5). This pattern was confirmed with the generalized linear mixed effects model, with bilinguals experiencing significantly more AV-integration than monolinguals (β = −2.42, SE = 0.85, Wald’s *z* = −2.85, *p* = 0.004). Tukey-adjusted pairwise comparisons (with significance at *p* < 0.05) reveal that this was the case for both late (β = −2.29, SE = 0.97, *z* = −2.36, *p* = 0.047) and early bilinguals (β = −2.55, SE = 0.95, *z* = −2.68, *p* = 0.020). As in the ANOVA, no difference was found between early and late bilinguals (β = −0.26, SE = 0.89, *z* = −0.29, *p* = 0.956). These findings suggest that bilinguals rely on visual information more than monolinguals when comprehending speech. Furthermore, the lack of a proficiency effect, as well as the fact that increased integration was found for early bilinguals (many of whom were dominant in English), suggests that the bilingualism effect is not due to the use of a less fluent language.

#### 3.1.2. Noise Condition

**Figure 4:**
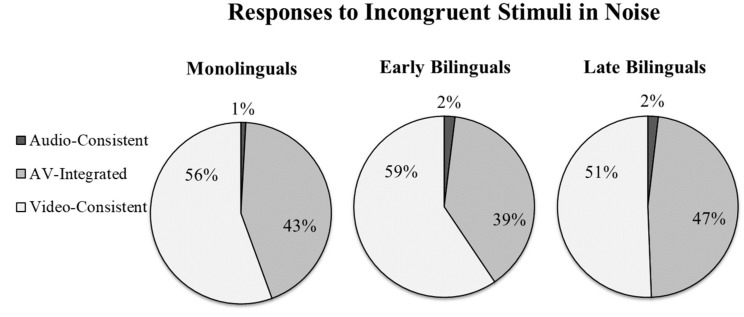
Percentage of auditory-consistent responses, AV-integrated responses, and video-consistent responses to incongruent stimuli in the Noise condition.

The ANOVA analysis revealed no main effects of Language Group (F(2,44) = 0.544, *p* = 0.584), Proficiency (F(1,44) = 0.02, *p* = 0.880), nor an interaction (F(2,44) = 0.24, *p* = 0.790). This result was confirmed by the generalized linear mixed effects model, with no significant difference between bilinguals and monolinguals (β = −0.06, SE = 0.58, Wald’s *z* = −0.11, *p* = 0.911) or between early and late bilinguals (β = −0.65, SE = 0.67, Wald’s *z* = −0.97, *p* = 0.331). As can be seen in Figure 4, when the auditory stimulus was sufficiently degraded, everyone relied heavily on the visual channel irrespective of language experience.

**Figure 5:**
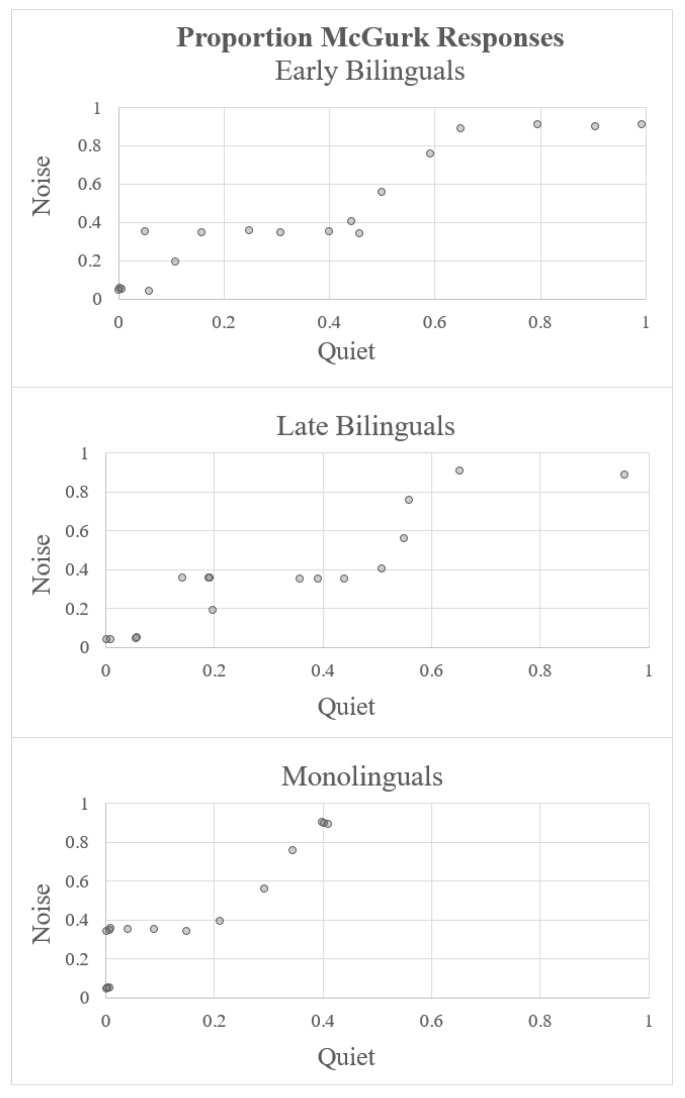
Relationship between the proportion of AV-integrated responses under noisy and quiet conditions. All data points were jittered by a random value between ±0.01.

In order to visually inspect the reliability of the language effect, we plotted the proportion of AV-integrated, McGurk percepts for each individual participant under noisy and quiet conditions (Figure 5). Participants were ranked according to the amount of integration experienced under the two conditions. These rankings were positively correlated, suggesting that there was consistency in susceptibility to McGurk effects within individuals (r = 0.299, t(49) = 2.19, *p* = 0.033). While there was substantial variability across individuals, monolinguals were consistently less likely to report AV-integrated responses compared to bilinguals under quiet conditions, as can be seen in Figure 5. 

### 3.2. Speech Perception Ability

Next, we investigate the question of whether the early and late bilinguals were worse overall at detecting speech, even when the audio was presented alone or with congruent visual stimuli. If both groups are indeed less accurate during speech perception, we cannot rule out the possibility that the bilingual increase in audiovisual integration is a compensatory mechanism. On the other hand, if no perceptual deficits are found for one or both of the bilinguals, it provides further support that bilingual experience can affect audiovisual integration independently of proficiency. 

The mean accuracies in each condition for audio-only and congruent audiovisual stimuli are presented in Table 2. Because participants’ responses were close to ceiling in the quiet condition (mean accuracy = 98.92%), we restricted our analyses to the noise condition. The ANOVA revealed significant main effects of Language Group (F(2,88) = 5.62, *p* = 0.005), with monolinguals outperforming bilinguals, and AV status (F(1,88) = 204.22, *p* < 0.0001), with participants performing better with congruent audiovisual stimuli than audio-only stimuli. There was no effect of self-reported proficiency (F(1,88) = 0.181, *p* = 0.671) and no interactions (all *p* > 0.1). Bonferroni-corrected pairwise comparisons (significance at *p* < 0.016) demonstrated that monolinguals were significantly more accurate than late bilinguals (F(1,56) = 10.38, *p* = 0.002), but not early bilinguals (F(1,62) = 2.53, *p* = 0.116). Early and late bilinguals did not differ from each other (F(1,58) = 3.83, *p* = 0.055). In all cases, there were significant main effects of AV status (all *p* < 0.001), but no effects of self-reported proficiency or interactions (all *p* > 0.1). These results provide a measure of proficiency and demonstrate that while late bilinguals experienced some deficit in comprehending speech under noisy conditions, early bilinguals and monolinguals performed equally well. These findings were confirmed with the generalized linear mixed effects analysis, with monolinguals outperforming bilinguals when collapsing across early and late learners (β = −0.40, SE = 0.14, Wald’s *z* = −2.87, *p* = 0.004). Tukey-adjusted pairwise comparisons (significance at *p* < 0.05) reveal that this effect of language group was driven by late bilinguals, who were significantly less accurate than monolinguals (β = −0.48, SE = 0.16, *z* = − 3.00, *p* = 0.008), whereas no differences were found between either early bilinguals and monolinguals (β = −0.32, SE = 0.16, *z* = −2.05, *p* = 0.101), or between the two bilingual groups (β = 0.16, SE = 0.15, *z* = 1.05, *p* = 0.545). All participants benefited from having congruent audiovisual stimuli as compared to audio-only (β = 1.50, SE = 0.12, Wald’s *z* = 12.97, *p* < 0.0001), and there were no interactions (all *p* > 0.2).

## 4. Discussion

The goals of the current study were two-fold. First, we sought to examine whether bilingual experience changes audiovisual processing by increasing reliance on visual information. Second, we investigated whether the bilingual increase in audiovisual integration arises from immediate deficits in perceiving speech sounds, or whether it is due to lasting differences in how bilinguals and monolinguals process sensory input. To explore these questions, we compared English-speaking monolinguals to early and late Korean-English bilinguals to determine how they perceived speech-sounds that were paired with either congruent or incongruent visual information. 

As predicted, bilingual participants were more likely to perceive fused McGurk-type sounds (e.g., /da/) when presented with incongruent auditory and visual information (e.g., hearing /ba/ but seeing /ga/). This suggests that relative to monolinguals, those with bilingual experience were more influenced by the visual channel. Critically, we find no evidence that this was especially the case for late bilinguals, as might be expected if the effect was solely a result of poor language comprehension. In fact, early bilinguals actually experienced the most McGurk-like percepts of the three language groups. Additionally, while late bilinguals were less accurate at identifying audio-only and congruent audiovisual speech relative to monolinguals, no such deficit was observed for the early bilinguals. Many of the early bilinguals were actually dominant in English, and yet this group still experienced greater audiovisual integration relative to their monolingual peers. We propose that beyond compensating for on-line difficulties in comprehending speech, early bilingual experience may fundamentally change how people attend to and subsequently perceive audiovisual information. 

It should be noted that the proposed bilingualism effect is independent of a potential proficiency effect. However, these factors have often been conflated in previous investigations with bilingual participants having lower proficiency than their monolingual counterparts. The present study contributes to our understanding of how language background affects audiovisual integration by comparing monolinguals to bilinguals who are either less or equally proficient relative to the monolinguals. This comparison allows us to examine the relative contributions of language proficiency versus bilingualism per se. Earlier, we proposed that early difficulties in acquiring and managing multiple languages may form habits of perceptual processing. These challenges, either as infants in relatively complex linguistic environments or as adults acquiring a new language, may lead to strategic attention to visual information that persists even after proficiency issues have been resolved. There are, however, additional explanations for this bilingual effect. For example, enhanced executive function in bilinguals may free up resources for binding sensory inputs. While the integration of audiovisual stimuli may appear to be done effortlessly, research shows that the McGurk effect is significantly reduced under high attentional demands [42]. When participants were forced to complete a concurrent auditory or visual secondary task, audiovisual integration was minimized, suggesting that attentional resources are required for effective cross-modal binding. As noted previously, there is evidence that bilingual experience can enhance executive function and thereby improve the ability to multitask. For example, Bialystok [43] found that bilingual children outperformed monolinguals on an audiovisual dual-task exercise. A greater attentional capacity to attend to both sensory channels could thus result in increased integration and likelihood of experiencing McGurk effects. 

The relevance of investigating the factors that influence sensory integration is highlighted by the fact that both methodological and individual differences can impact the prevalence of McGurk effects. For instance, in a systematic examination of AV-integration utilizing 14 different stimuli and 165 participants, Magnotti and Beauchamp [44] found that the rate of McGurk responses ranged from 17% to 81% across stimuli, and that the average standard deviation across individuals was approximately 40% from the mean. Such variability is also apparent when comparing the average proportion of McGurk percepts in the current study (28% across language groups) with that of a study utilizing the same stimuli (van Wassenhove, Grant, Poeppel [41] with approximately 70%). This variability is likely due to methodological differences between the two studies. For instance, (1) the present study included trials in noise and a single onset asynchrony while van Wassenhove et al.’s did not; (2) our stimuli were presented at 60 dB as opposed to 70 dB; (3) we utilized ear buds as opposed to speakers; and (4) we had six response options as opposed to three. In addition to the substantial methodological differences, the different rates of McGurk responses may have resulted from the relatively small sample size combined with the high variability that has traditionally been found across individuals.

Some individuals nearly always hear fused percepts and others never hear them [45]. These individual differences can even be traced to neurological activity with those who are susceptible to McGurk effects showing increased activation of the superior temporal sulcus (STS), which is an area associated with cross-modal binding [46]. The likelihood of experiencing McGurk effects has also been associated with individual differences in sensory functions, such as how much sensory noise there is during encoding [44] and how temporally close two inputs must be to be perceived as one [47]. Differences have also been observed among clinical populations, such that children with autism spectrum disorder are less influenced by visual input and thus less likely to experience McGurk-like percepts relative to typically developing children [48,49]. Other factors such as lip-reading ability [50], age [51], and familiarity with the speaker [52] have also been shown to affect susceptibility to the McGurk effect. Most pertinent to the current investigation, the listener’s native language may also play a role. While robust McGurk effects have been found in many different languages such as Italian, Spanish, German, and Thai [15,53,54], some differences among languages and cultures have been observed. For instance, there is evidence that both Japanese and Chinese speakers experience weaker McGurk effects relative to native English speakers, possibly due to differences in cultural norms, such as those pertaining to how appropriate it is to look at interlocutors’ faces [16,55]. Hebrew speakers have also been found to experience fewer McGurk effects relative to English speakers, likely due to differences in linguistic characteristics such as voice-onset-time [56]. The current study demonstrates that bilingual experience is another factor that can contribute to the degree of audiovisual integration in speech. Furthermore, it is possible that the effect of bilingualism may interact with other factors. For instance, we may expect bilingualism to have a smaller effect for native speakers of languages such as Japanese who are already less susceptible to the McGurk effect. Given the high variability across individuals and groups, future research would benefit from exploring the bilingualism effect with larger sample sizes that account for both individual and sociolinguistic differences.

In addition to examining the robustness of the phenomenon, future research could include investigations into whether this bilingual increase in audiovisual integration is specific to speech or generalizes to non-speech stimuli. The latter hypothesis is made plausible by evidence suggesting that comprehension of speech recruits processes shared by both speech and non-speech stimuli [57]. Saldaña and Rosenblum [8] found that McGurk-like illusions could be elicited using cello sounds, which were perceived differently depending on whether the participant simultaneously viewed bowing or plucking. If the effect of bilingualism generalizes beyond speech stimuli, we may observe increased integration in such non-linguistic tasks as well. It would also be of interest to explore whether the effect of bilingualism is specifically due to increased weighting of visual stimuli, or rather a more general tendency to bind sensory information rather than attend to a single channel. After all, factors other than bias towards the visual modality can influence the likelihood of binding inputs. For instance, the probability of two inputs coming from the same source (i.e., “causal inference”) is an important predictor of audiovisual integration [58]. As noted previously, one factor that contributes to the perception of a common source is the temporal relationship between the two stimuli [47], and there is evidence suggesting that the timing required for two stimuli to be perceived as simultaneous differs for native and non-native speech [59]. If bilinguals are more likely to bind sensory inputs (due to factors such as greater causal inference), audiovisual integration may be bidirectional, with bilinguals also experiencing more influence of auditory stimuli on visual perception [60]. Such investigations would enhance our understanding of the scope of the bilingual effect and shed light on the extent to which experience in one domain can have broad-reaching consequences. 

## 5. Conclusions

In conclusion, our data suggest that linguistic experience plays a role in determining the extent to which individuals bind sensory information. We find that bilinguals are more prone to audiovisual integration, even in the absence of deficits in speech comprehension. This greater reliance on visual information leads to changes in how sounds are perceived, for both early and late bilinguals. Today, it is estimated that at least half of the world’s population is multilingual [61] and with continued globalization, more people join these ranks every day. It is therefore critical to gain a comprehensive view of the consequences of knowing more than one language, ranging from high-level societal impact to low-level perceptual changes. Our findings provide evidence for the powerful role that experience plays in shaping cognition. Just as working as a London taxi driver can enlarge the hippocampus [62] and playing video games can enhance visual selective attention [63], we find that bilingual experience can influence basic cognitive processes, altering the very way we perceive the world around us. 

## Figures and Tables

**Table 1:** Age, age of acquisition (AoA), and proficiency.

	Monolinguals	Early Bilinguals	Late Bilinguals	Mono vs. Early	Mono vs. Late	Early vs. Late
*N*	17	18	16	-	-	-
Age	21.71 (2.42)	20.44 (2.20)	21.38 (2.00)	n.s.	n.s.	n.s.
English AoA	0.24 (0.44)	3.44 (2.04)	9.07 (1.83)	*	*	*
English Proficiency	9.76 (0.44)	9.31 (0.87)	8.62 (0.93)	n.s.	*	*
Korean AoA	-	0.94 (1.21)	0.47 (1.06)	-	-	n.s.
Korean Proficiency	-	8.39 (1.83)	9.13 (0.85)	-	-	n.s.

The three columns on the right present group comparisons between monolinguals and early bilinguals, between monolinguals and late bilinguals, and between early bilinguals and late bilinguals. For group comparisons, asterisks represent a significant difference by *t*-test at *p* < 0.05. Non-significant comparisons are represented by “n.s.”.

**Table 2:** Accuracy for congruent stimuli.

	Monolinguals	Early Bilinguals	Late Bilinguals
	(*N* = 17)	(*N* = 18)	(*N* = 16)
**Audio-Only**			
Quiet	0.99 (0.02)	0.99 (0.02)	0.97 (0.04)
Noise	0.31 (0.19)	0.22 (0.15)	0.15 (0.14)
**Audio-Visual**			
Quiet	1.00 (0.00)	0.99 (0.03)	0.99 (0.02)
Noise	0.79 (0.17)	0.75 (0.15)	0.70 (0.14)

Accuracy for audio-only stimuli and audiovisual stimuli in the congruent condition.
